# Sox9 is required for prostate development and prostate cancer initiation

**DOI:** 10.18632/oncotarget.531

**Published:** 2012-07-02

**Authors:** Zhenhua Huang, Paula J Hurley, Brian W Simons, Luigi Marchionni, David M Berman, Ashley E Ross, Edward M Schaeffer

**Affiliations:** ^1^ Department of Urology, Johns Hopkins Medical Institutions, Baltimore, MD, USA; ^2^ Department of Pathology, Johns Hopkins Medical Institutions, Baltimore, MD, USA; ^3^ Department of Oncology, Johns Hopkins Medical Institutions, Baltimore, MD, USA

**Keywords:** Sox9, prostate, development, cancer, initiation

## Abstract

Prostate cancer is one of the most common malignancies and the second leading cause of death from cancer in men. The molecular mechanisms driving prostate carcinogenesis are complex; with several lines of evidence suggesting that the re-expression of conserved developmental programs plays a key role. In this study, we used conditional gene targeting and organ grafting, to describe conserved roles for the transcription factor Sox9 in the initiation of both prostate organogenesis and prostate carcinogenesis in murine models. Abrogation of Sox9 expression prior to the initiation of androgen signaling blocks the initiation of prostate development. Similarly, Sox9 deletion in two genetic models of prostate cancer (TRAMP and Hi-Myc) prevented cancer initiation. Expression profiling of Sox9-null prostate epithelial cells revealed that the role of Sox9 in the initiation of prostate development may relate to its regulation of multiple cytokeratins and cell adherence/polarity. Due to its essential role in cancer initiation, manipulation of Sox9 targets in at-risk men may prove useful in the chemoprevention of prostate cancer.

## INTRODUCTION

Prostate organogenesis is initiated when androgen signaling induces epithelial proliferation, invasion, and bud formation. Mapping of the precise androgen-initiated programs of early prostate growth has illuminated roles for many conserved developmental programs[1] and implicated the reactivation of them in malignant prostatic growth[2, 3].

Sox9 is a developmental transcription factor vital for the regulation of sex determination[4], cartilage development[5], intestinal differentiation[6] and adult progenitor cell pool maintenance[7, 8]. We identified Sox9 as one of the earliest molecules expressed in the primordial prostate[1], predating even the expression of Nkx3.1, a transcription factor and classical marker of prostate lineage. Sox9 is embryonic lethal and thus prior studies have utilized an Nkx3.1 driven Cre-recombinase to conditionally deleted Sox9 in the prostate. This conditional deletion of Sox9 demonstrated a requirement for Sox9 in ventral prostate differentiation[9], implying a functional role for Sox9 in prostate development.

Herein, we demonstrate that Sox9 expression precedes and modulates expression of Nkx3.1. We utilized a tamoxifen (TAM)-inducible ERCre-Sox9^flox/flox^ conditional knockout system[10] and prostate organ grafting[11] to precisely delete Sox9 throughout development and adulthood. With this approach we demonstrate a requirement for Sox9 in the initiation of prostate organogenesis and define a role for Sox9 in progenitor cell maintenance. In sum, we identify Sox9 as a critical mediator of the prostate epithelial lineage.

Given the key role of Sox9 in the initiation of prostate organogenesis and the observation of high Sox9 levels in premalignant prostatic intraepithelial neoplasia (PIN) lesions in human prostate cancers[1], we investigated whether Sox9 was also integral to prostate cancer initiation. Utilizing the same TAM-inducible, organ-grafting system in combination with two genetic models of prostate cancer, we found that loss of Sox9 blocked cancer formation in both the TRAMP and Hi-Myc model systems. Together, these studies implicate Sox9 as a critical factor required for the initiation of prostate organogenesis and carcinogenesis and highlight an opportunity to develop therapies directed at Sox9 and its targets.

## RESULTS

### Sox9 expression occurs earlier than Nkx3.1 in the developing prostate

**Figure 1:**
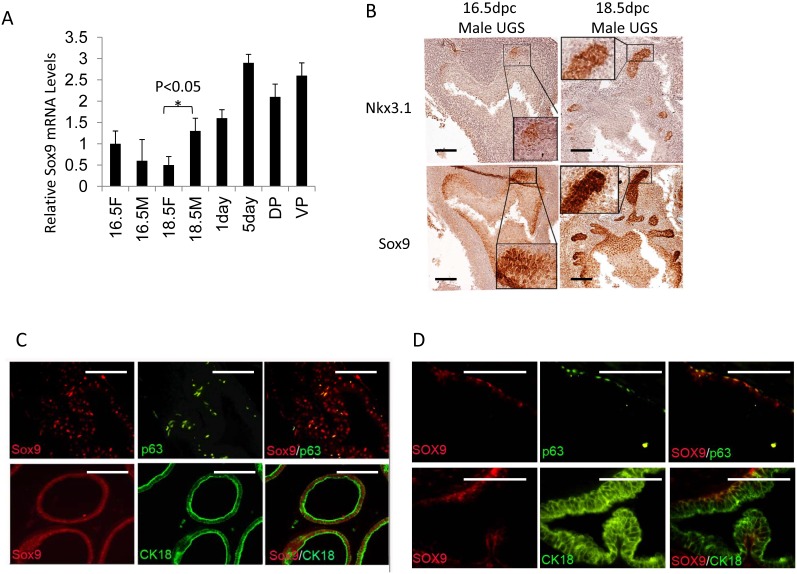
Sox9 and Nkx3.1 expression in UGS and adult prostate A. Sox9 mRNA levels in male (M) and female (F) UGS at different ages (16.5F or 16.5M =16.5-dpc female or male UGS, 18.5F or 18.5M =18.5-dpc female or male UGS, 1day or 5day=1 or 5 day prostate, DP=adult dorsal prostate, VP=adult ventral prostate). B. Sox9 and Nkx3.1 expression in male UGS at different ages. C/D. p63 (green), CK18 (green), Sox9 (red) expression in adult mouse (C), and adult human prostate (D) as detected by IF. Scale bars = 100μM.

In investigating the androgen-initiated programs of prostate development, we identified Sox9 induction in early prostate organogenesis[1]. By 16 days post-conception (dpc) in the mouse and 10 weeks in the human, initiation of prostatic development occurs[12]. In response to circulating androgen, Sox9 mRNA and protein are induced (Fig.1A, Supplemental Fig. 1). At 16.5-dpc, Sox9 is diffusely expressed in UGS epithelial (UGE) cells while Nkx3.1, another early marker of prostate induction, is expressed in only a limited number of UGE cells (Fig.1B). Serial sectioning and staining suggests a sub-population of epithelial cells co-express Sox9 and Nkx3.1 (Fig.1B). By 18.5-dpc, Sox9 is enriched at the tips of invading epithelial buds (Fig.1B). In adult mouse prostate, Sox9 is expressed in both basal and luminal epithelial cells (Fig.1C) with the proximal prostate lobes expressing higher Sox9 levels than the distal tubules (Supplemental Fig. 2B). Sox9 is predominantly expressed in basal cells in the adult human prostate as determined by co-localization with the basal cell marker p63 (Fig. 1D). Rare/occasional Sox9 positive cells can be seen in the luminal compartment of the human prostate (Supplemental Fig. 2C). Sox9 protein expression in the mouse is enriched during prostate regression following castration (Supplement Fig. 2A,B) Overall, these data suggest very early, androgen-mediated induction of Sox9 in the developing prostate.

### DHT increases Sox9 expression in the UGS by up-regulation of FGF2 in the UGM and subsequent activation of Erk1/2

**Figure 2:**
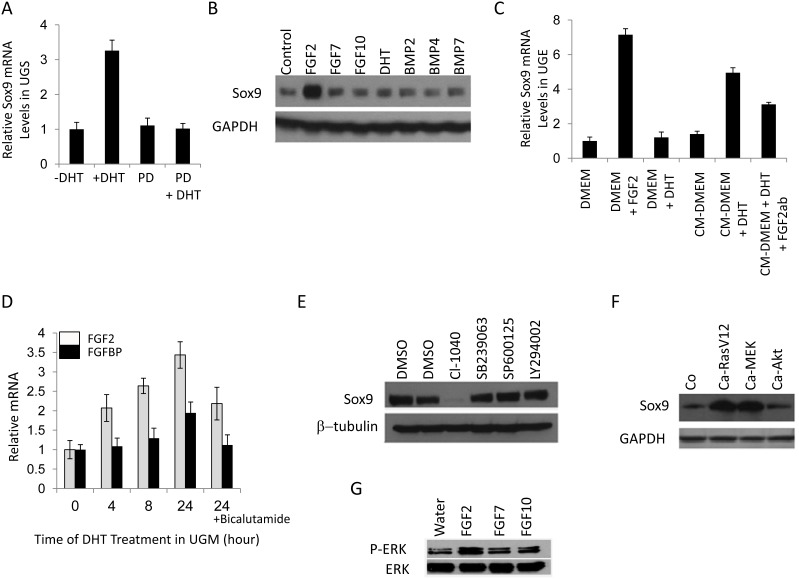
Sox9 expression in UGS is regulated by DHT and fibroblast growth factor receptors (FGFRs) A. DHT induced Sox9 mRNA in UGS is dependent on FGFR. 16.5-dpc male UGS were maintained in organ culture with or without 10^−8^ M DHT and 10 μM PD173074 (PD, an FGFR inhibitor) for 24 hours. The mRNA levels were then evaluated by RT-PCR. B. FGF2 significantly increases Sox9 protein in mouse UGE, which was isolated and cultured as described in Methods. UGE were incubated in UGE medium without growth factors for one day, and then various growth factors were added into the medium. Two days later, the treated cells were analyzed by immunoblotting. C. Conditional medium from UGM containing DHT induces Sox9 mRNA in UGE. Isolated mouse UGE cells were cultured with UGE medium for three days and with DMEM without growth factors for another day. UGE cells were incubated with different media for 18 hours, and then Sox9 mRNA levels in treated cells were analyzed by RT-PCR. CM-DMEM, conditional medium for UGM without DHT; CM-DMEM-DHT, conditional medium for UGM with DHT; Fgf2ab, Fgf2 antibody. D. DHT induces Fgf2 and FgfBP in UGM. UGM isolated from 16.5-dpc UGS were cultured in UGM medium as described in Methods. After incubation in serum-free medium for one day, UGM cells were treated with DHT for different times. The Fgf2 and FgfBP mRNA levels of treated cells were analyzed by RT-PCR. E. Sox9 protein expression in UGS is regulated by Erk. Male UGS (16.5-dpc) were isolated and organ cultured in CI-1040 (MEK inhibitor), SB239063 (p38 inhibitor), SP600125 (JNK inhibitor), or LY294002 (Akt inhibitor) for 48 hours, and then analyzed by immunoblotting. F. Constitutively activated RAS (Ca-RasV12) and MEK (Ca-MEK) but not Akt (Ca-Akt) up-regulate Sox9. UGE cells were infected with retroviruses expressing constitutively activated RAS, MER, and Akt. Three days later, the cells were analyzed by immunoblotting. G. UGE cells were starved for 1 day in UGE medium without growth factors and were then incubated with FGF2, FGF7 FGF10. 16 hrs later, the cell lysates were analyzed by immunoblotting for total and phospho-Erk1/2.

Sox9 expression in UGS is induced in the presence of androgen but it is less clear whether its expression is controlled directly or involves additional paracrine growth pathways (Fig. 2A). Indeed, Sox9 expression does not change upon exposure of UGE to DHT (Fig. 2B,C), suggesting that paracrine growth factors released from the UGM upon androgen stimulation may be responsible for Sox9 induction. To determine which ligands mediate this, we examined the ability of multiple growth factors to induce Sox9 in the UGE. FGF2 significantly increased Sox9 mRNA and protein levels in the UGE (Fig. 2B,C). Accordingly, inhibition of the FGF receptor signaling attenuated DHT induced Sox9 expression (Fig. 2A). Consistent with a paracrine signaling model of androgen-induced Sox9 expression mediated by FGF2, we observed DHT-initiated induction of FGF2 and FGF binding protein (FGFBP) in the UGM (Fig. 2D). In addition, while DHT-containing UGM conditioned media could induce Sox9 expression in UGE, this was attenuated in the presence of an FGF2 blocking antibody (Fig.2C). Together this implies that androgens mediate UGM induction of FGF2 with paracrine up-regulation of Sox9 in the UGE. To elucidate pathways downstream of FGF2 induced Sox9 expression, we cultured UGS in the presence of inhibitors of MEK, p38, JNK and AKT. Of these, only MEK inhibition abrogated Sox9 expression (Fig.2E). To compliment this, we infected UGE cells with retroviruses expressing active forms of RAS, MEK or AKT. Sox9 induction was seen with activated RAS and MEK, but not AKT (Fig.2F). Concordant with these findings, incubation of UGE with FGF2 (but not FGF7 or 10) activated the MAP kinase pathway as assayed by Erk1/2 phosphorylation (Fig 2G). Together, this supports that early in prostate organogenesis, androgen induced mesenchymal FGF2 regulates Sox9 induction in the UGE through the Erk1/2 pathway.

### Sox9 is essential for prostate initiation in organ culture

Mice deficient in Sox9 are embryonic lethal, thereby limiting their use to examine prostate development. Utilizing a prostate epithelia-specific Nkx3.1 promoter to drive Cre recombinase, others have described a partial defect in prostate development upon Sox9 loss(9). Because we noted earlier and stronger expression of Sox9 than Nkx3.1 in UGS (Fig.1B), we considered the possibility that Sox9 has an earlier and more essential role in prostate development than previously appreciated. To delete Sox9 at the earliest, inductive phases of prostate development (14.5-16.5-dpc) when UGS tissue expresses androgen receptor and is responsive to androgen, we combined renal grafting of UGS tissues[13] with a TAM-inducible ERCre system[10]. These ERCre^+/+^Sox9^flox/flox^ (ER-Sox9^flox/flox^) mice allow for deletion of Sox9 during multiple phases of prostate development and adulthood with a low dose of TAM that does not affect the prostate[14].

**Figure 3:**
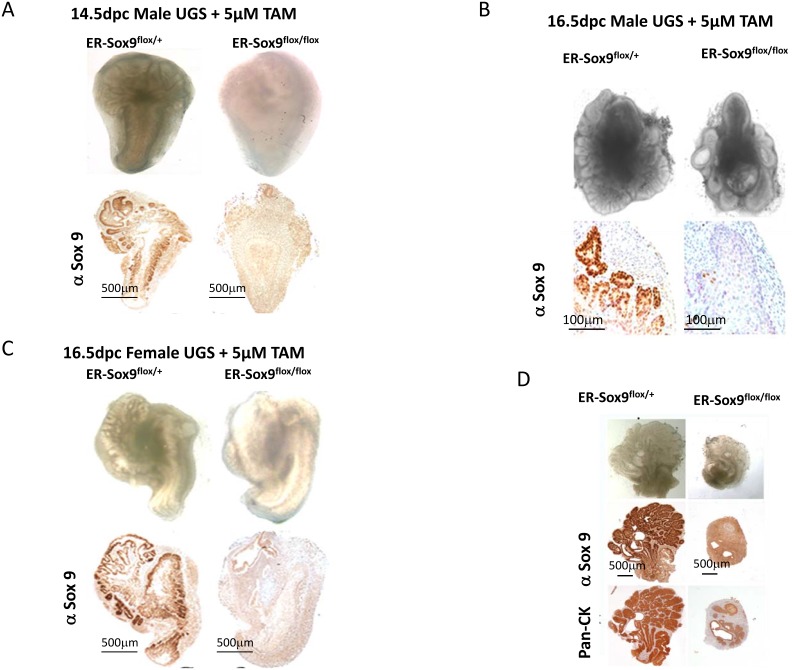
Sox9 is essential for prostate budding A-C. Sox9 is essential for prostate initiation. Male (14.5-dpc) (A), male (16.5-dpc) (B), and female (16.5-dpc) (C) ER-Sox9^flox/+^ or ER-Sox9^flox/flox^ UGS were cultured in organ culture medium with 5 μM TAM and DHT. On the 10^th^ day, the UGS were imaged, processed, and examined by IHC for Sox9 expression. D. Sox9 mediates prostate branching. Ventral prostates were isolated from day-1 postnatal ER-Sox9^flox/+^ and ER-Sox9^flox/flox^ mice, and cultured with 5 μM TAM. After 10 days, the prostates were imaged and examined by IHC for Pan-CK and Sox9 expression. Scale bars = 100μM, unless otherwise noted.

In culture, androgens can induce prostate organogenesis in 14.5-dpc male UGS with the formation of prostate buds (Fig.3A). However, when Sox9 was deleted with TAM, 14.5-dpc male ER-Sox9^flox/flox^ UGS failed to initiate prostate organogenesis (Fig. 3A).When Sox9 expression was abrogated in older (16.5-dpc) tissue, after *in vivo* exposure to endogenous androgen from the testes had occurred, small numbers of prostate buds appeared (Fig. 3B). To confirm that the small degree of bud formation was from endogenous androgen, we cultured androgen-naïve 16.5-dpc UGS from genotypic females since they possess androgen receptors and are androgen responsive. In these tissues, Sox9 deletion inhibited prostate initiation as seen in 14.5-dpc UGS (Fig.3C). Together, these results suggest that the requirement for Sox9 in prostate organogenesis occurs prior to the onset of androgen exposure. To further explore the relationship between Sox9 expression and androgen exposure, we cultured the 14.5-dpc UGS in different temporal combinations of TAM and DHT. Budding was abrogated only when Sox9 expression was deleted prior to androgen exposure (data not shown). Similarly, in post natal day1 prostate tissue, where androgen exposure has occurred *in utero*, bud elongation occurs but is attenuated (Fig. 3D). Thus prior to androgen exposure, Sox9 plays an essential role which allows for the initiation of prostate organogenesis.

### Sox9 is required for UGS development in vivo

**Figure 4:**
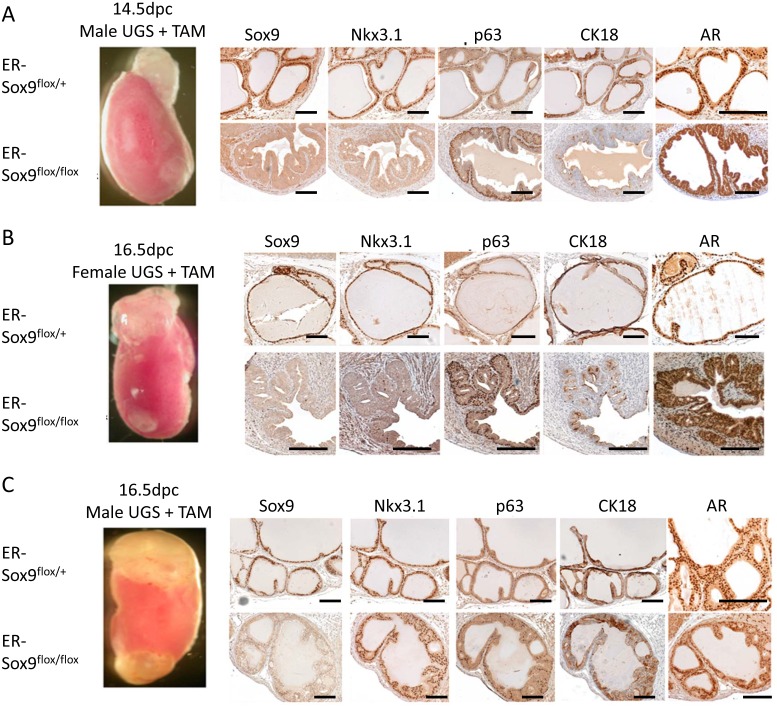
Sox9 is required for prostate differentiation in vivo A-C. Male (14.5-dpc) (A), male (16.5-dpc) (B), and female (16.5-dpc) (C) ER-Sox9^flox/+^ and ER-Sox9^flox/flox^ UGS were incubated in organ culture with 5 μM TAM and 10^−8^ mM DHT for 7 days and then grafted into the kidneys of SCID mice. After two months, the grafts were examined by IHC for Sox9, Nkx3.1, p63, and CK18 and AR expression (A-C). Scale bars = 100 μM

Renal grafting of UGS tissue parallels normal *in vivo*, *in situ* prostate development and allows for a longitudinal assessment of prostate development and subsequent maturation in mice with genetically lethal mutations[15]. We combined renal grafting with a TAM-inducible conditional knockout to investigate the functions of Sox9 in later phases of prostate development. ER-Sox9^flox/flox^ and ER-Sox9^flox/+^ UGS were first treated in organ culture with TAM to delete Sox9 and then grafted under the renal capsules of SCID mice. After two months, Sox9-null UGS from 14.5-dpc male (Fig.4A) and 16.5-dpc female UGS (Fig. 4B) failed to develop into prostatic tissue, whereas similarly aged heterozygous ER-Sox9^flox/+^ UGS exposed to similar conditions differentiated into normal prostatic tissue (Fig. 4A, 4B). Consistent with the culture observations (Fig. 2B), male 16.5-dpc ER-Sox9^flox/flox^ UGS grafts exhibited an intermediate phenotype in which a small amount of normal prostate tissue developed with normal expression of the prostate markers Nkx3.1, p63, CK18 and androgen receptor (AR) (Fig.4C, Supplemental Fig 3). In androgen-naïve UGS grafts, IHC staining demonstrated complete Sox9 deletion and loss of Nkx3.1 and Ck18, but not AR or the basal cell marker p63 (Fig. 4A, 4B, Supplemental Fig 3). Thus, in the androgen-naïve state, Sox9 expression is required for the induction of androgen-mediated prostate organogenesis. Once androgen exposure occurs, this requirement for Sox9 is lifted, suggesting that Sox9 acts to direct development of the prostate epithelial lineage.

### Sox9 is necessary for prostasphere formation and self-renewal

**Figure 5:**
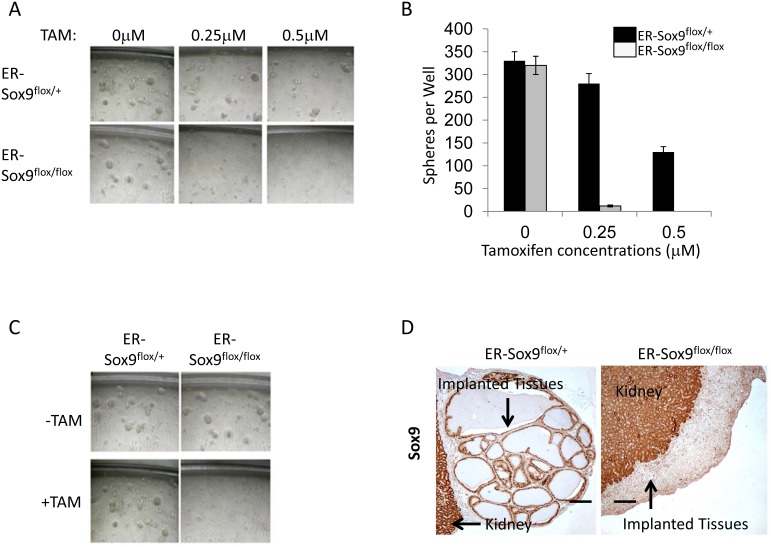
Sox9 is required for prostasphere formation, self-renewal in vitro, and regeneration in vivo A-B. Sox9 is necessary for prostasphere formation. Prostate epithelial cells from ER-Sox9^flox/+^ or ER-Sox9^flox/flox^ adult mice were cultured in matrigel with or without TAM. On the 10th day, images of prostaspheres were acquired (A) and the colonies of prostaspheres (>40 μm) were counted (B). C. Sox9 is indispensable for prostasphere self-renewal. Ten-day first-generation prostaspheres were treated with or without TAM. passaged to form second-generation prostaspheres and imaged ten days later. D. Sox9 is essential to regenerate into prostate tubules *in vivo*. Ten-day, TMA-treated, first-generation prostaspheres were digested/implanted with UGM into renal capsules. Grafts were examined 2 months later and stained Sox9 expression with IHC. Formation and renewal assays were carried out 5 times.

Sphere-forming cells represent a subset of stem/progenitor cells necessary for normal differentiation and/or carcinogenesis in many tissues, including prostate[16]. Wild-type and ER-Sox9^flox/+^ prostate epithelial cells retained the ability to form prostaspheres even in the presence of TAM (280±45) (Fig. 5A and B). In contrast, Sox9 deficient epithelial cells formed very few spheres compared to Sox9^flox/+^ epithelial cells ~4% (12±4 spheres). Prostasphere regeneration, which can occur over multiple generations, has been used to identify the regenerative potential of prostate epithelial cells. While Sox9 deficient prostaspheres remained viable (Supplemental Fig.4), they were not capable of self renewal (Fig.5C). In addition, Sox9-null prostaspheres completely lost the ability to develop into prostate glands *in vivo* (Fig.5D). These observations identify a critical requirement for Sox9 in prostate stem cell maintenance and differentiation.

### Sox9 is dispensable in adult prostate maintenance

Because systemic Sox9 deletion is lethal in embryogenesis[17] and adulthood, we investigated the role of Sox9 in the maintenance of the prostate. Specifically, we implanted ER-Sox9^flox/flox^ and ER-Sox9^flox/+^ UGS under the renal capsules of SCID mice and two months later, after normal development had occurred, deleted Sox9. After 5 months the glands were histologically similar, expressed normal prostate markers and weighed equivalent amounts (0.25±0.04 mg vs 0.26±0.03) (Supplemental Fig. 5). These data indicate that Sox9 is not required to maintain a mature prostate gland.

### Sox9 is required for Nkx3.1 re-expression in regenerating adult prostates

**Figure 6:**
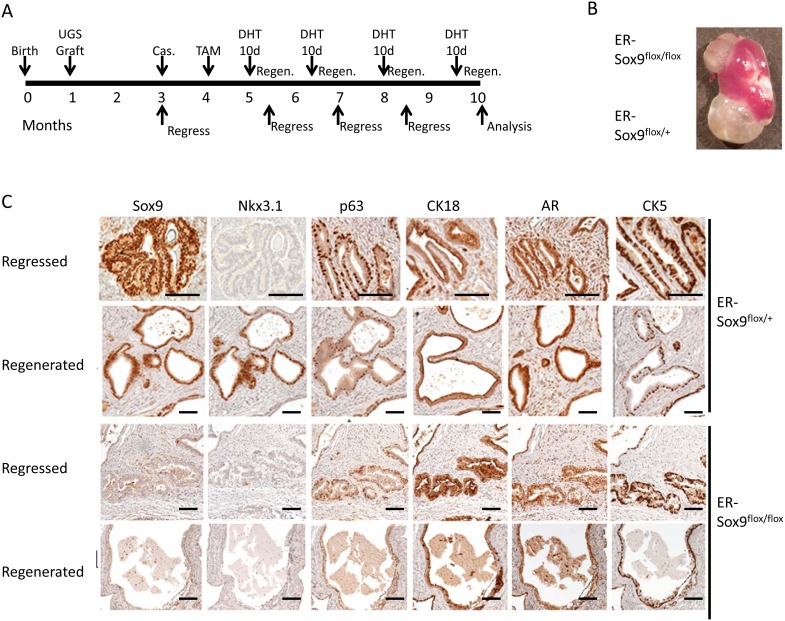
Sox9 is dispensable for adult prostate regeneration A. Timeline for serial prostate regression-regeneration assay in castrated mice. Castration regeneration experiments were carried out with seven sets of UGS grafts. Both ER-Sox9^+/+^ and ER-Sox9^flox/+^ tissue were used as controls with no difference seen. Castration regeneration was also carried out for two rounds of hormone replacement with subcutaneous (vs oral) DHT (50mg/kg) with yielded similar results (Data not shown). B. Gross images of grafts after 10 months. C. Cycled grafts were examined for Sox9, Nkx3.1, p63, CK18, AR, and CK5 expression by IHC. Scale bars = 100 μM

Prostate glands retain the ability to regenerate after androgen ablation-a process postulated to depend on a subset of epithelial cells with regenerative potential and signals from their stromal microenvironment[16, 18]. To determine whether Sox9 participates in prostate regeneration, we implanted sets of ER-Sox9^flox/flox^ and ER-Sox9^flox/+^ UGS under contra-lateal renal capsules of SCID mice. Following grafting, mice were castrated, given TAM to ablate Sox9 expression, and then cycled for four rounds of prostate regression-regeneration (Fig. 6A). The Sox9-null regenerated tissues were slightly smaller than their controls (0.20±0.07mg vs 0.24±0.07mg, Fig.6B) but not significantly (p=0.09). Consistent with the normal castration process[19], Nkx3.1 expression was lost after castration in both ER-Sox9^flox/flox^ and ER-Sox9^flox/+^ grafts (Fig. 6C). While Nkx3.1 expression was restored in ER-Sox9^flox/+^ tissues upon androgen re-exposure, Sox9-null tissues failed to express Nkx3.1 after 4 rounds of androgen cycling (Fig. 6C). Otherwise, the regenerated Sox9 null glands had a normal distribution of prostate epithelial cell markers (AR, Ck18 Ck5 and p63) (Fig.6C, Supplemental Fig6). In sum, when the epithelial-stromal relationship is preserved in the mature prostate, regeneration is not grossly affected by the loss of Sox9. Although Nkx3.1 re-expression was lost, we detected no additional abnormalities over the 10 months.

### Sox9 is required for prostate carcinogenensis in animal models

Embryonic programs can be reactivated in cancers, with some postulating that a subset of undifferentiated cells with stem cell properties may drive oncogenesis[20]. Given the role of Sox9 in the initiation of prostate organogenesis, we sought to determine its role in the initiation of prostate carcinogenesis.

The transgenic adenocarcinoma of the mouse prostate (TRAMP) model is a well-established model which exhibits remarkable similarities to human prostate cancer[21]. Precancerous mPIN lesions develop beginning around 4 months and by 8 months are replaced by invasive adenocarcinoma[21] (Supplemental Fig.7). In mPIN lesions, we detected high levels of Sox9 expression that declined as invasive tumors developed (Supplemental Fig.7) which mimicked our observations in human prostate cancer cases[1]. Thus to explore the role of Sox9 in prostate carcinogenesis, we deleted Sox9 in TRAMP prostates using the Probasin-Cre4 transgene but noted incomplete abrogation of Sox9 expression (Supplemental Fig.8). Therefore, to obtain complete deletion, we generated TRAMP-ER-Sox9^flox/flox^ and TRAMP-ER-Sox9^flox/+^ prostate grafts in SCID mice. To control for any unforeseen side effects of limited TAM exposure, TRAMP-ER-Sox9^flox/+^ and TRAMP-ER-Sox9^flox/flox^ prostates were implanted in the same mice in contra-lateral kidneys.

**Figure 7:**
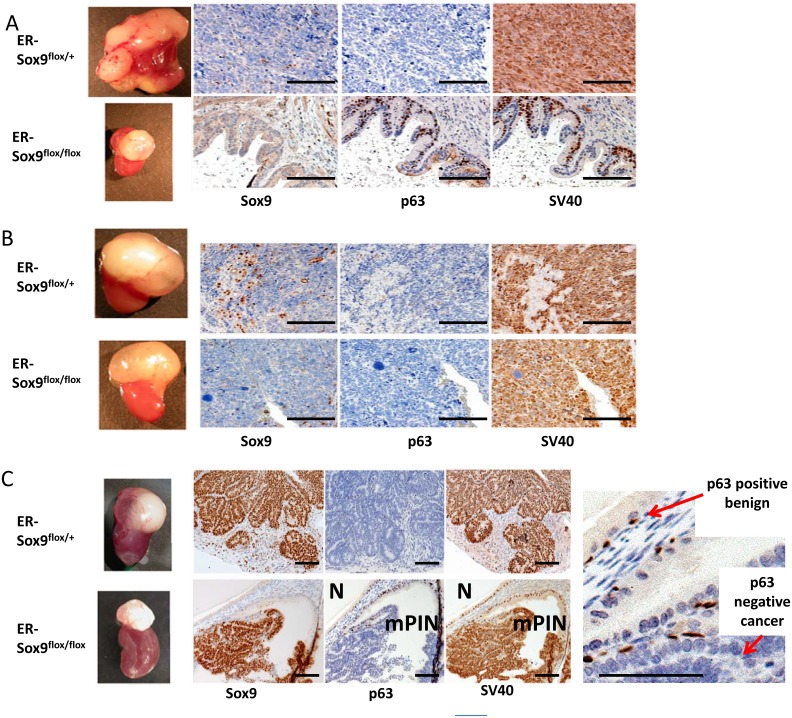
Sox9 is essential for prostate cancer initiation A. Sox9 deletion (early) before the development of mPIN inhibits prostate carcinogenesis in the TRAMP model. 6 pairs of TRAMP prostate grafts grown under the renal capsule of SCID mice were treated with TAM 1.5 months after grafting and 8 months later weighed, processed and examined for Sox9, p63, and SV40 expression by IHC. B. Sox9 deletion (late) after the development of mPIN does not prevent prostate carcinogenesis in the TRAMP model. 5 pairs of TRAMP grafts were grown for 3 months, treated with TAM and weighed, processed and examined by IHC 7 months later. C. Early, incomplete Sox9 deletion resulted in hybrid Sox9 glands with both normal and mPIN/cancer architecture. Sox9 null glands expressed p63 and were normal (N) while Sox9 positive glands lacked p63 expression and resembled mPIN/cancer (mPIN). Scale bars = 100 μM

In this system, grafted prostates developed mPIN and invasive cancer lesions along a timeline similar to prostates in TRAMP mice. 1.5 months after grafting, mice were exposed to TAM and aged 10 months (Supplemental Fig.9-T1). Whereas all 6 TRAMP-ER-Sox9^flox/+^ prostates developed into invasive adenocarcinoma (Fig.7A), the 6 TRAMP-ER-Sox9^flox/flox^ prostates retained normal prostate glandular structure with p63-positive and Sox9-negative expression (Fig.7A). Wet weights of prostate tissues from TRAMP-ER-Sox9^flox/+^ were greater than those from TRAMP-ER-Sox9^flox/flox^ tissues (2.9±0.5 gm vs 0.25±0.1 gm respectively, p=0.01) (Fig.7A). The tissues from the TRAMP-ER-Sox9^flox/flox^ grafts expressed SV40 large T antigen, confirming the appropriate genotype (Fig.7A). In sum, the (early) absence of Sox9 expression in the prostate prevents progression to mPIN and cancer in the TRAMP model.

To determine whether carcinogenesis could be abrogated at later time points, we delayed Sox9 deletion until after the development of mPIN (around 4 months following implantation) (Supplemental Fig.9-T2). With this, invasive adenocarcinoma occurred in both TRAMP-ERCre-Sox9^flox/flox^ and TRAMP-ER-Sox9^flox/+^ tissues (at 7 months in 5 sets of grafts (Fig.7B)). The tumors were histologically indistinguishable with a mean size (1.8±0.3 vs 2.14±0.6) that was not different (p=0.07) (Fig.7B). Together, this suggests that Sox9 deletion after mPIN initiation cannot prevent progression from mPIN to invasive carcinoma.

Interestingly, in hybrid glands (generated with low dose TAM exposure), tumors consistently appear but are slightly smaller. Sox9-negative cells retained normal architecture and remained p63-positive while Sox9-positive cells histologically resembled mPIN and were p63-negative (Fig. 7C, Supplement Fig.9-T3). Thus Sox9 is necessary for the initiation of prostate cancer in TRAMP mice.

To determine if Sox9 is necessary for cancer initiation in other models prostate cancer, we generated Hi-Myc prostates conditionally deficient for Sox9 expression. In Hi-Myc mice, mPIN lesions develop at around 4–6 months and progress to invasive cancers at around 6–8 months[22]. We assessed the role of Sox9 in Hi-Myc prostate carcinogenesis through renal grafting as described above. Sox9 deletion prevented mPIN/tumor formation in Hi-Myc prostates in contrast to Sox9 expressing glands which all developed invasive cancer (Supplemental Fig.9-T4, 10).

### Sox9 targets in prostate epithelial cells are enriched for multiple keratins and calcium-related genes

Given the roles of Sox9 in development and cancer we sought to identify a Sox9-dependent transcriptome. Expression profiling of Sox9 deficient epithelial cells derived from prostaspheres (Table S2,S3) uncovered Sox9-dependent expression of multiple cytokeratins including Krt5 and Krt14, two established cytokeratin markers of basal subtypes of prostate epithelial cells. Gene expression was confirmed by RT-PCR. Interestingly, Nkx3.1 was not down-regulated upon Sox9 deletion which, along with the absence of a Sox9 consensus DNA binding site in the Nkx3.1 promoter, suggests indirect regulation of Nkx3.1 by Sox9.

In addition, we found Sox9 dependent induction of Col17a1 (5.2 fold) which has been shown previously to be involved in cellular adhesion and orientation. Coinciding with this, via analysis of functional annotation, we identified enrichment of molecular themes modulated by Sox9 including those for cell adhesion/polarity and stem cell pathways (Table S4).

## DISCUSSION

Sox9 is highly induced during androgen-driven organogenesis of the prostate, with increased Sox9 expression seen prior to induction of Nkx3.1, the earliest known marker of prostate differentiation. Sox9 plays a critical role in the earliest inductive phase of prostate organogenesis. In contrast, Sox9 is not required for glandular maintenance during hormonal (androgen) cycling when contact with its stromal microenvironment is preserved. During prostasphere regeneration, where epithelial cells lack stromal cues, Sox9 is essential for epithelial regeneration from prostaspheres. Lastly, the abrogation of Sox9 from adult prostate tissue in two genetic models of prostate cancer results in a complete block of carcinogenesis. Together, these data suggest a conserved role for Sox9 in maintaining the identity/lineage of a prostate epithelial cell throughout different phases of both physiologic and pathologic growth.

### Androgen induces Sox9 in UGS via mesenchymal-dependent FGF signaling

Prostate development is androgen dependent with the initial androgenic effects occurring through mesenchymal androgen receptor signaling. Paracrine signaling by FGFs and other growth factors induce prostate epithelial cell lineage and bud outgrowth, ductal branching, and prostatic differentiation[23]. Our results indicate that Sox9 is essential in the early phase of this mesenchymal–epithelial interaction during prostate organgenesis. We observe UGM dependent induction of Sox9 in the UGE through epithelial FGFR–MEK-ERK activation. The precise mechanisms downstream of Erk1/2 that induce Sox9 in the prostate are not known but our expression analysis (Tables S2-4) suggests that several canonical Erk1/2 output signals including myc and fos may play a role. In other systems, including murine intestinal development, Sox9 has been reported to be regulated by alternate signaling cascades including the Wnt pathway[24]. We have generated catenin null prostates which are deficient in canonical Wnt signaling and have not observed Wnt dependent expression of Sox9 (B.Simons personal communication). Thus in prostate development, the initial androgen induction of Sox9 is through Fgf2 signaling, a growth factor also overexpressed in prostate cancer [25, 26].

### Sox9 expression in UGS is required for the initiation of prostate development

During initial molecular phases of prostate development, Sox9 expression precedes that of Nkx3.1, suggesting that studies utilizing Nkx3.1-driven Cre deletion of Sox9 may have underestimated the early role of Sox9 in prostate organogenesis[9]. Indeed, when Sox9 is deleted prior to androgen exposure, UGS tissue fails to develop along the prostate lineage. Once physiologic exposure to androgen occurs, the absolute requirement for Sox9 is abated, suggesting that Sox9 is crucial for the initiation UGE/prostate epithelial lineage. In support of this, Sox9 does not significantly affect graft size in the mature prostate during androgenic cycling. Interestingly, Nkx3.1 expression is absent after 4 rounds of androgenic cycling and this may suggest a more subtle role for Sox9 in maintaining gland identity when prostate epithelial cells are in contact with their normal mesenchymal/stromal microenvirnment.

When prostate epithelial cells are isolated from the stromal microenvironment, they are critically dependent on Sox9 for prostatic epithelial stem/progenitor cell pool maintenance as seen by an inability of Sox9 deleted cells to form and self renew prostaspheres. Recent work has demonstrated that Sox9 marks adult stem cell populations in several organs of endodermal origin (i.e., liver, pancreas, and intestine)[7], and also contributes to the self-renewal and repair of these organs. We provide evidence that Sox9 plays a similar role in the endodermally derived prostate epithelial cell. Thus in the absence of the normal prostate microenvironment, Sox9 is required for prostate epithelial cell maintenance. Also these data further support the hypothesis that the stromal microenvironment plays a key role in maintaining self renewing stem cell niches[27, 28] and suggests that when this microenvironment is altered (for example, through the loss or alteration of mesenchymal signals by oncogenic factors), stem cells may become more dependent on pathways allowing for cell autologous growth[28]. Such a hypothesis may explain the necessity for Sox9 in cancer formation and its dispensability in normal tissue maintenance.

### Sox9 is required for the initiation of prostate carcinogenesis in two genetic models of the disease

In the TRAMP model, which recapitulates multiple phases of prostate cancer, we found Sox9 expression highest in mPIN lesions a finding that parallels our previous observation in human prostate lesions. When Sox9 is deleted from TRAMP prostate grafts before mPIN formation, mPIN/tumors do not form; this is in contradistinction to Sox9-heterozygous TRAMP grafts, which develop invasive cancers. Consistent with this requirement for Sox9 during cancer initiation, deletion of Sox9 after the mPIN stage did not prevent TRAMP grafts from developing into aggressive tumors. Sox9 is also required for cancer initiation in the Hi-Myc prostate cancer model. This block in carcinogenesis suggests the possibility that Sox9 maintains a prostate epithelial lineage that is responsive to additional oncogenic stimuli and thereby allows for the initiation of prostate carcinogenesis.

One key feature of prostate epithelial lineage is the expression of different cytokeratins by different epithelial sub-types. Expression profiling demonstrates dramatic Sox9 regulation of cytokeratins Ck5 and Ck14 two markers of basal epithelial cells in the prostate, supporting a role for Sox9 in maintaining prostate epithelial lineage. In addition, gene expression based pathway enrichment analysis identified Sox9 as modulating cell polarity and adherence. Col17a1 is a novel Sox9 target that has an established role in cellular adhesion and polarity in the skin[29]. Its role in the prostate remains to be fully unraveled, but its potential role in prostate epithelial adhesion and orientation are intriguing.

Prior work suggests Sox9 plays a role in prostate cancer progression[30]. However, abrogation of cancer initiation in two models of prostate cancer with Sox9 loss suggests another key role for Sox9 in prostrate carcinogenesis. In fact, it is in these PIN lesions in both mice and humans that Sox9 expression is highest. How Sox9 expression allows for pathway deregulation in the transition to human prostate cancer remains unclear and is an active focus of our current research.

In summary, Sox9 is an essential transcription factor for the initiation of prostate organogenesis and carcinogenesis. Expression profiling of Sox9-null prostate epithelial cells reveals a role for Sox9 in the regulation of prostate epithelial identity through its regulation of multiple cytokeratins and/or calcium-related proteins. This work defines a novel permissive role for Sox9 in cancer initiation and suggests that manipulation of Sox9 targets in at-risk men may prove useful in the chemoprevention of prostate cancer.

## MATERIALS AND METHODS

### Mouse strains

C57BL/6, CB17 SCID/SCID, Rosa26ERCre, and Transgenic Adeno-carcinoma of the Mouse Prostate (TRAMP) mice were purchased from The Jackson Laboratory. Hi-Myc and Probasin-Cre4 mice were purchased from the NCI Mouse Repository at Frederick. All procedures were reviewed and approved by the Animal Care and Use Committee at Johns Hopkins University.

### Primary cell isolation and culture

Urogenital sinus epithelium (UGE) and mesenchyme (UGM) were isolated from urogenital sinus (UGS) of C57BL/6 murine embryos as described[11].

### Plasmid construction and virus production

Retroviral constructs Rasv12 (Ca-Rasv12), MEK (Ca-MEK) and Akt (Ca-Akt) were provided by Dr. Lodish (Massachusetts Institute of Technology). Retroviral particles were made and used as previously described[31].

### RNA preparation and quality assessment

Total RNA was isolated using the RNeasy kits from Qiagen[32]. Reverse transcription (GE Healthcare) and Quantitative reverse transcription-polymerase chain reaction was performed with SYBR Green.

### Organ culture

UGS from male and female fetuses were prepared [33] and cultured on 0.45 μM Millicell CM filters (Millipore Corp, Bedford,MA) in organ culture media (Supplemental methods).

### Prostasphere assay in matrigel or in suspension

Adult prostate tissues were isolated, minced and incubated in collagenase(1mg/ml) and DNase(1mg/ml). Cells were washed, cultured overnight and isolated into single cells and passed in Matrigel as described[34, 35]. Viability was assayed with Calcein AM dye (Invitrogen).

### Western blot assay

Treated cells or organs were harvested with ice-cold radioimmunoprecipitation assay (RIPA) buffer and analyzed by Western blot as described[36].

### Immunohistochemistry and immunofluorescence

Immunohistochemistry and Immunofluorescence was performed on sections of paraffin-embedded tissues as described[1]. Antibodies listed in Supplemental Table 1.

### Tamoxifen (TAM) and Dihydrotestosterone (DHT) administration

1 g TAM was dissolved in 2 ml ethanol and 18 ml peanut oil to reach a concentration of 5 mg/100 μl. TAM was orally given 5 mg/mice daily. DHT administration was carried out similarly (Dose 1.5 mg/mice /day).

### Renal grafts

Renal grafting of UGS, Prostaspheres and adult prostate tissue were performed under kidney capsule as described[34].

### Microarray Hybridization

Sox9 deficient epithelial cells were generated as above (*Prostasphere assay*). Total RNA was analyzed using a Bioanalyzer 2100 (Agilent Technologies, Santa Clara, CA, USA). RNA samples were amplified, labeled, and hybridized to whole Mouse[1] microarrays, (Agilent Technologies). Data was processed as described[1], and functional themes were obtained from Gene Ontology, KEGG and msigDb [37]. Gene expression data is available from Gene Expression Omnibus (GSE35419).

### Statistical Analysis

Results are shown as mean and standard deviations. Comparisons were made with Student's t-test (two tailed, paired). P values of <0.05 were considered statistically significant.

## Supplementary Figures and Tables




