# Supplementary material for: Sox9 is required for prostate development and prostate cancer initiation
Source: Oncotarget. 2012 Jul 2;3(6):651–63. doi: 10.18632/oncotarget.531 (PMC3442290; doi:10.18632/oncotarget.531)
Supplement: Supplementary file 1 [file oncotarget-03-651-s001.pdf]

Sox9 is required for prostate development and prostate cancer initiation - Huang et al

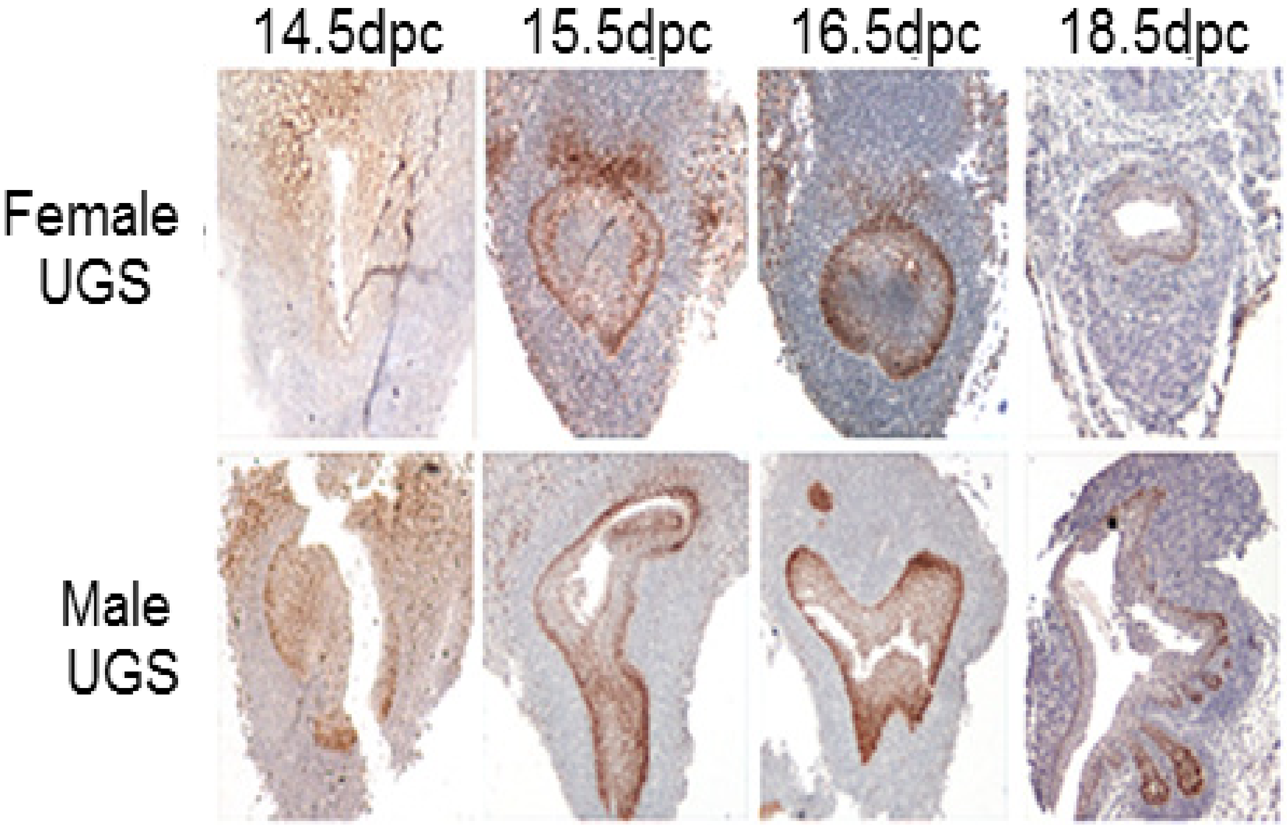

Supplemental Figure 1: Sox9 expression in mouse UGS. Female and male mouse UGS at different ages were examined for Sox9 expression by IHC.

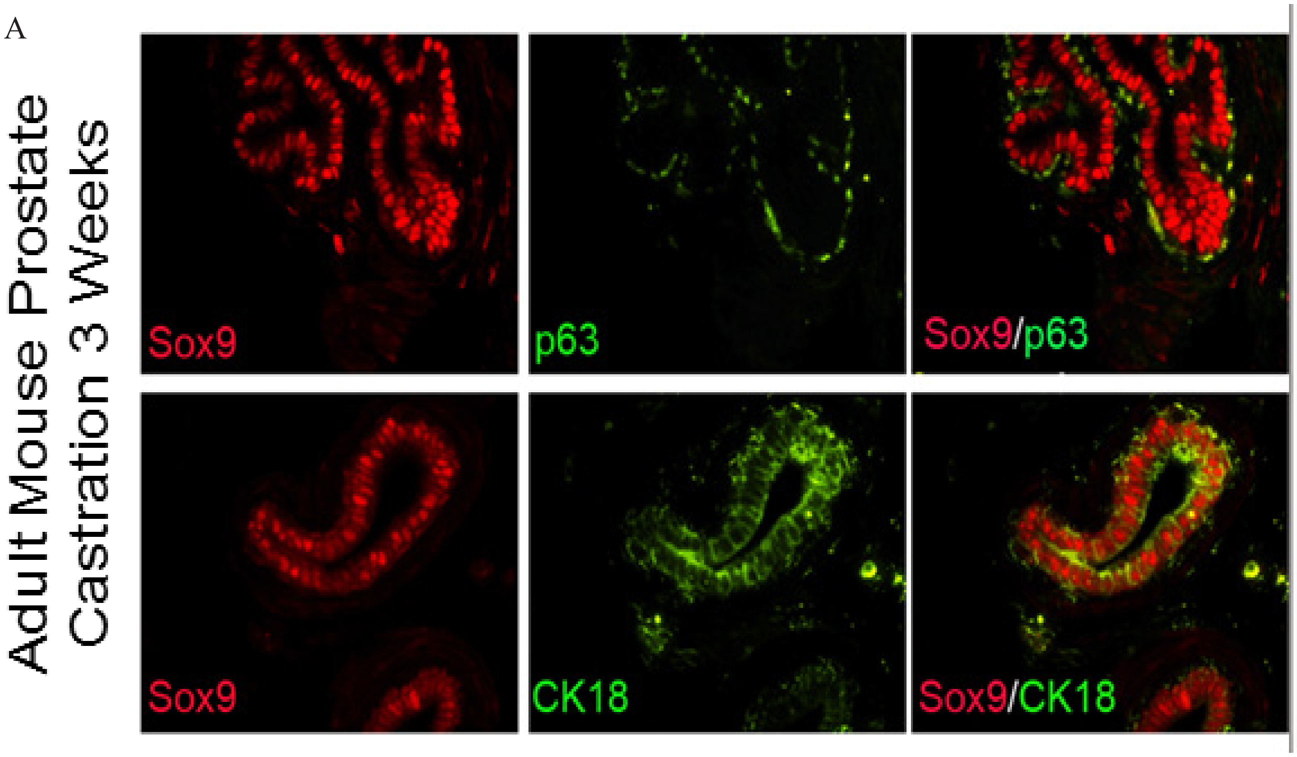

B

C

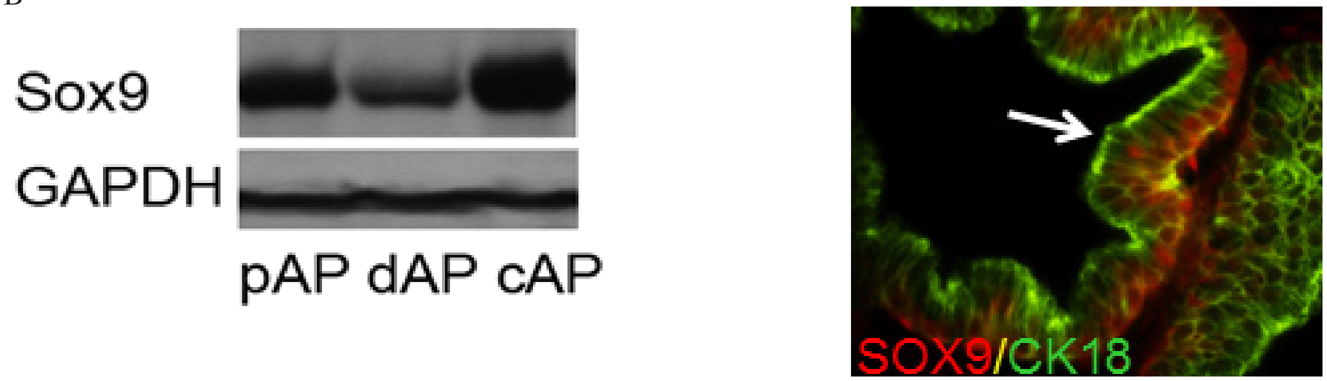

Supplemental Figure 2: Sox9 expression in adult and castrated mouse prostate and in human prostate. A. Sox9, p63, and CK18 expression in the prostate of castrated adult mice was detected by IF. B. Adult mouse anterior prostates (AP) were isolated and cut into two parts: proximal (pAP) and distal (dAP). AP (cAP) were isolated 19 days following castration. Sox9 expression in pAP, dAP, and cAP was analyzed by immunoblotting. C. Sox9 and CK18 expression in normal human prostate tissue was analyzed by IF.

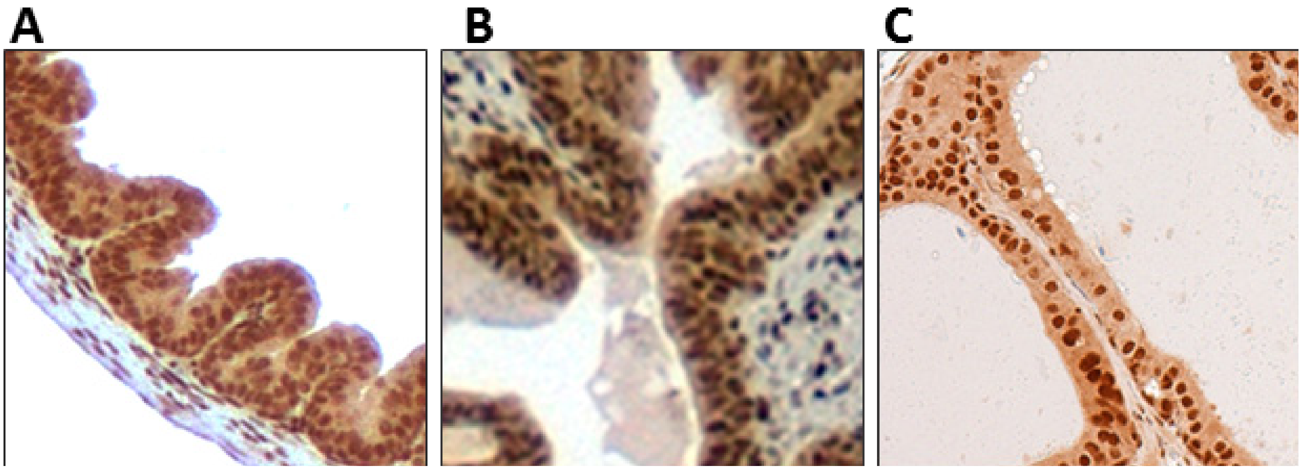

Supplemental figure 3: High power images of AR staining in in vivo prostate. Male (14.5-dpc) (A), male (16.5-dpc) (B), and female (16.5-dpc) (C) ER-Sox9$^{flox/flox}$ UGS were incubated in organ culture with 5 µM TAM and 10^{-8} mM DHT for 7 days and then grafted into the kidneys of SCID mice. After two months, the grafts were examined by IHC for AR expression. High power images are

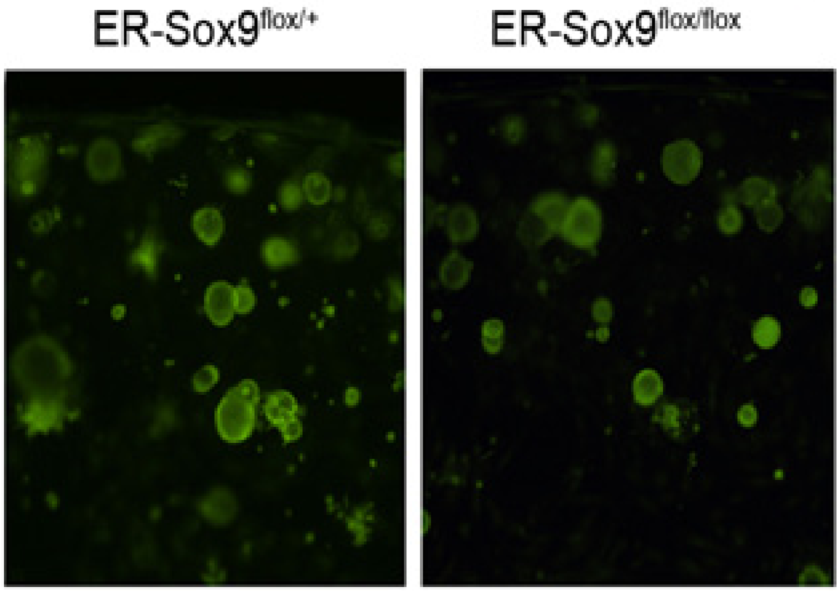

Supplemental Figure 4: TAM-treated prostasphere viability. 10-day prostaspheres were treated with 0.5 μM TAM for 6 additional days, and then incubated with 1.5 μM Calcein AM dye (Invitrogen) for 30 min. Uptake of green-fluorescent calcein indicates viability.

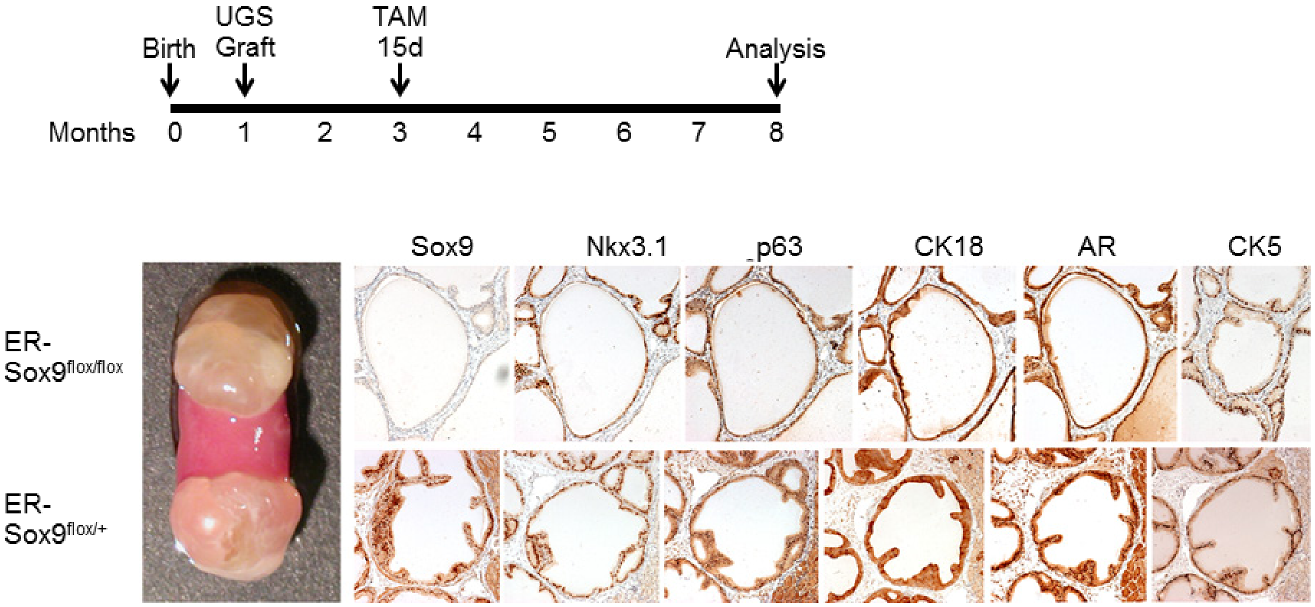

Supplemental Figure 5: Sox9 is dispensable in adult prostate maintenance. A. Adult prostate maintenance experimental timeline. B. Grafted tissues were photographed at the end of the 8th month. Sox9, Nkx3.1, p63, CK18, AR, and CK5 expression in the grafted tissue was assayed by IHC.

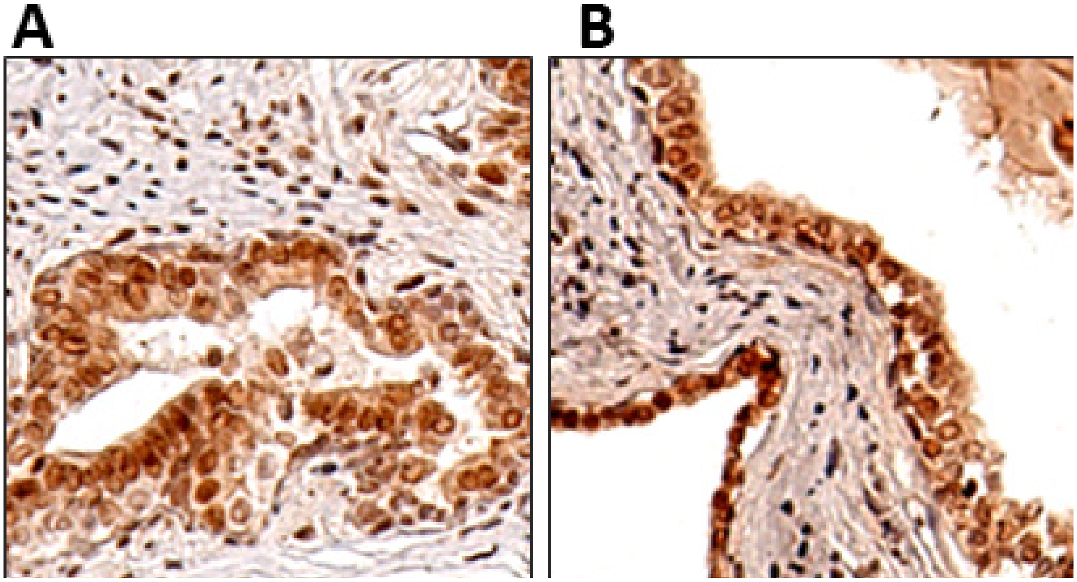

Supplemental Figure 6: Sox9 is not required for AR expression in the cycling prostate graft. High power images of ER-Sox9$^{flox/flox}$ prostate grafts that had been cycled 4 times as in Figure 6. A. High power of regressed graft stained for AR by IHC. B. High power of regenerated graft stained for AR by IHC.

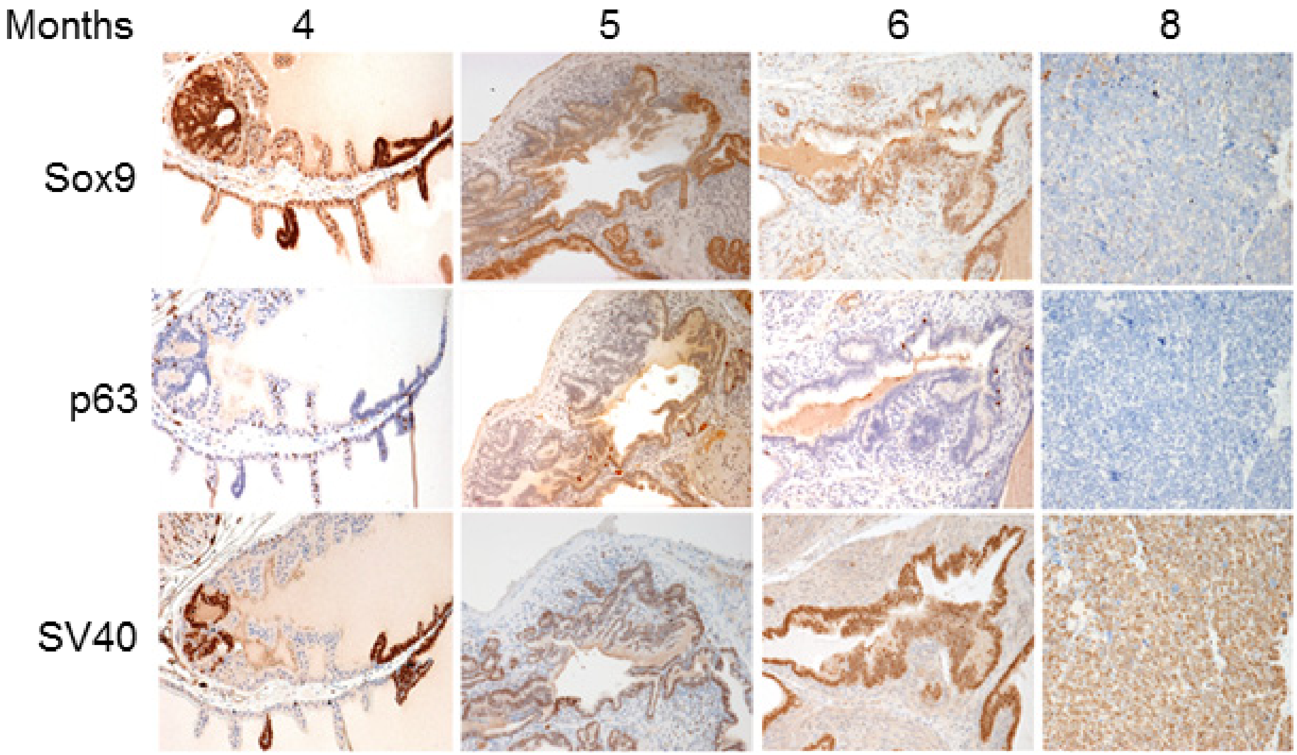

Supplemental Figure 7: Sox9 and p63 expression in TRAMP prostates at various time points. The process of carcinogenesis in wild type TRAMP mice characterized by Sox9, p63, and SV40 staining.

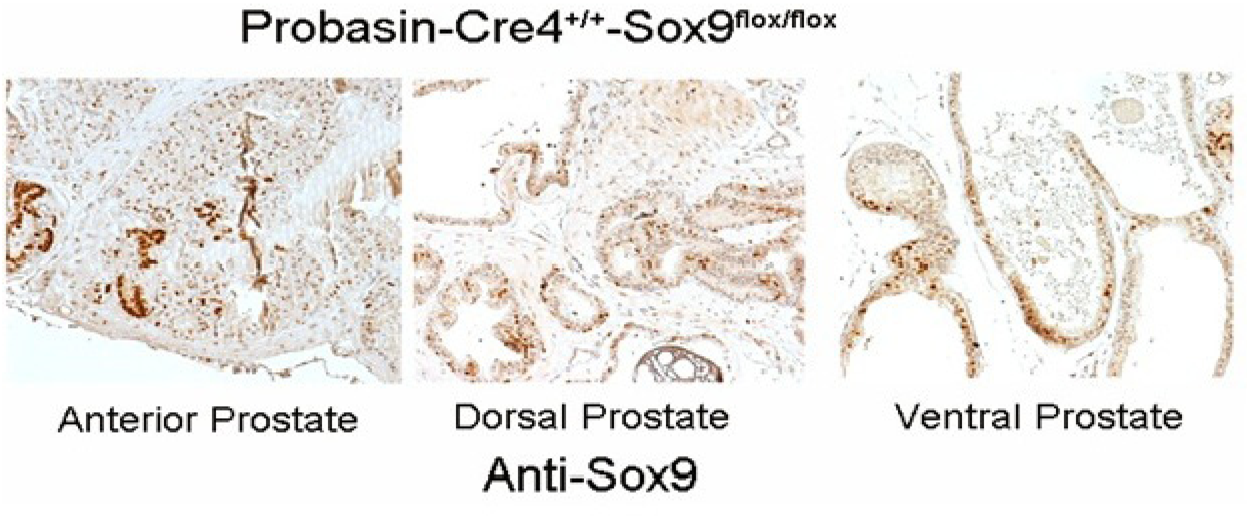

Supplemental Figure 8: Sox9 was incompletely deleted in Probasin-Cre+/+Sox9flox/flox mice. Probasin-Cre4 mice were cross with $Sox9^{flox/flox}$ to produce $Probasin\text{-}Cre^{+/+}Sox9^{flox/flox}$ mice. After 4 months, the mice were scarified and the prostate tissues were subjected to IHC staining. Hybrid grafts similar to Figure 7C were noted.

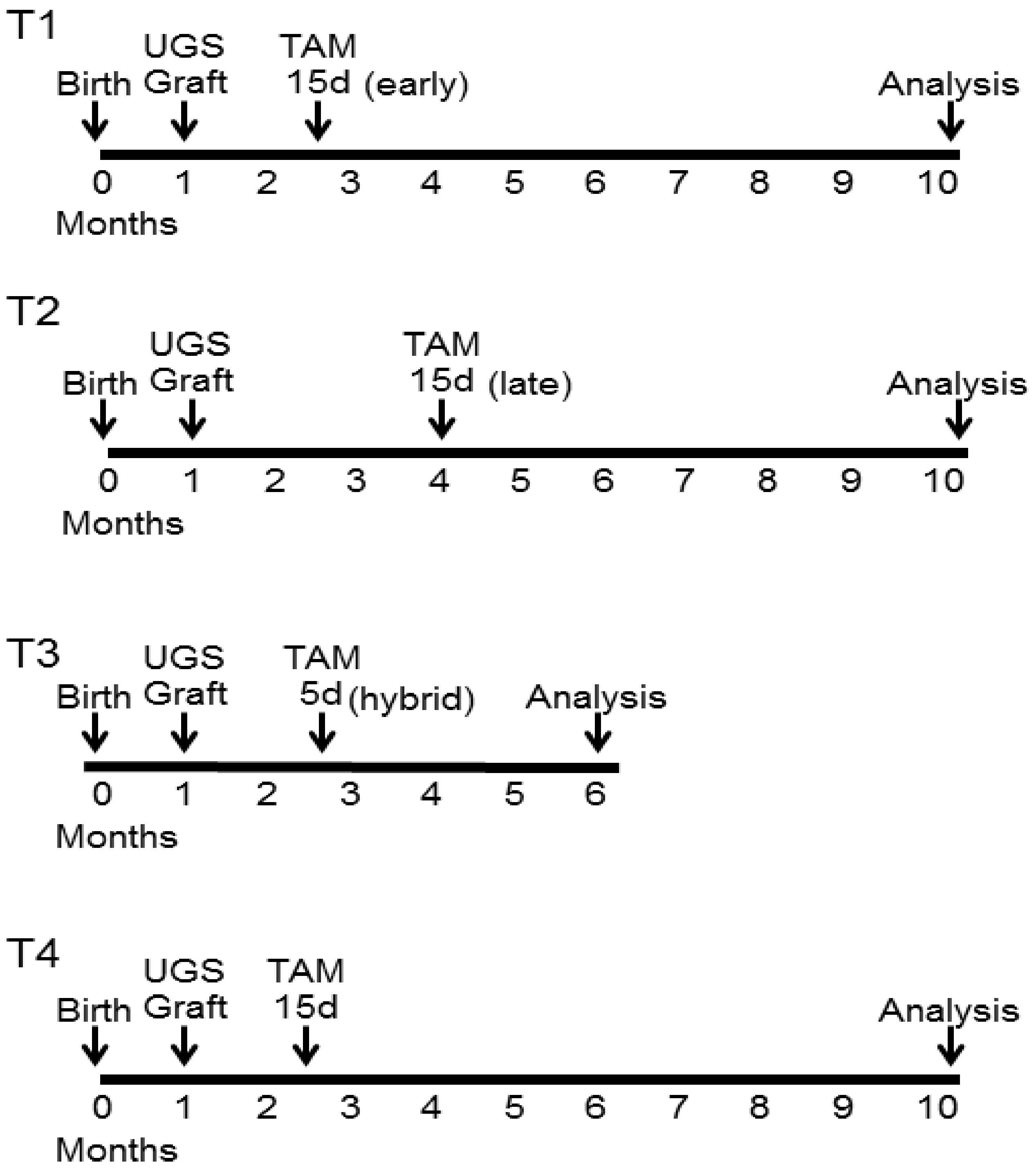

Supplemental Figure 9: Timelines for graft implantation and Tamoxifen induced deletion of Sox9 in TRAMP and Hi-Myc mice. T1. Timeline for the experiments of 15-day TAM treatment at 1½ months (early) in TRAMP mice.
T2. Experimental timeline for 15-day TAM treatment at the 4th month (Late) in TRAMP mice.
T3. Experimental timeline for 5-day TAM treatment at 1½ months (hybrid).
T4. Experimental timeline for 15-day TAM treatment at 1½ months in Hi-Myc mice.

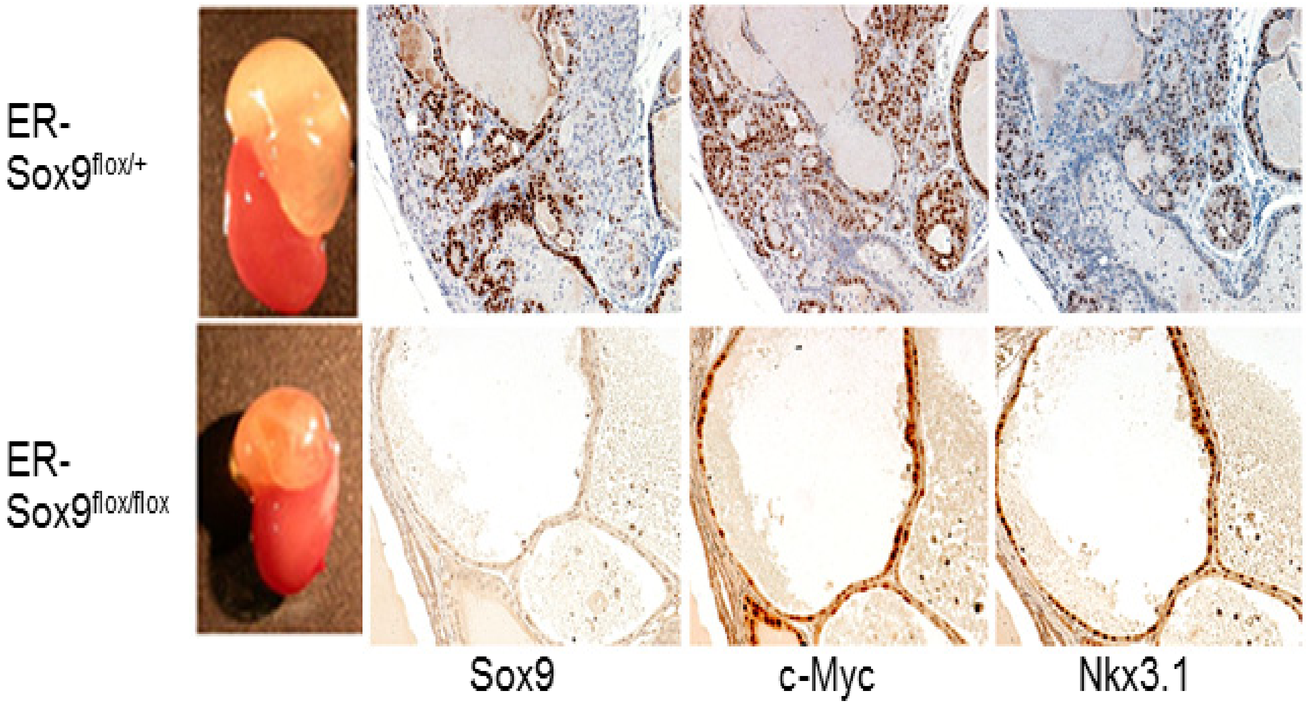

Supplemental Figure 10: Sox9 deletion prevents carcinogenesis in Hi-Myc prostate. Hi-Myc prostate grafts grown under the renal capsule of SCID mice were treated with TAM 1.5 months after grafting for 15 days. Tissues of TAM-treated Hi-Myc prostate tissues were stained with Sox9, c-Myc, and Nkx3.1. Nine sets of Hi-Myc x ER-Sox9$^{flox/+}$ or ER-Sox9$^{flox/flox}$ were generated with results consistent with images shown.
